# Characterizing Response Capacity of Healthcare-Associated Infection/Antimicrobial Resistance Programs — US, 2019–2022

**DOI:** 10.1017/ash.2024.281

**Published:** 2024-09-16

**Authors:** Nijika Shrivastwa, Penelope Strid, Maroya Walters, Kiran Perkins, Joseph Perz, Kelly Walblay, Patricia Kopp, Muhammad Salman Ashraf, Tisha Mitsunaga, Carolyn Stover, Kelley Garner, Jennifer C. Hunter

**Affiliations:** Centers for Disease Control and Prevention; Chicago Department of Public Health; South Carolina Department of Health and Environmental Control; University of Nebraska Medical Center; California Department of Public Health; Tennessee Department of Health; Arkansas Department of Health

## Abstract

**Background:** Since 2009, the CDC has invested in nationwide outbreak response capacity through Healthcare-associated Infections and Antimicrobial Resistance (HAI/AR) Programs in public health departments. The unpredictable nature of outbreaks requires public health programs to be able to scale operations and adapt strategies to effectively respond to emerging challenges, as demonstrated by the COVID-19 pandemic. This analysis characterizes HAI/AR Programs response capacity in scalability, adaptability, and technical expertise. **Method:** We reviewed data from HAI/AR Programs in 50 state, 6 local, and 2 territorial health departments (August 2019–December 2022). HAI/AR responses were defined as specific public health actions to assess an acute risk and prevent further harm in the context of a confirmed or possible healthcare outbreak; responses were categorized as involving novel or targeted multi-drug resistant organisms (nMDROs), COVID-19, and HAIs or infection control breaches. Descriptive statistics were used to analyze reported responses in three domains: scalability (number of responses per year), adaptability (number of pathogens and healthcare facility types involved in responses), and technical expertise (number of responses involving onsite or remote infection control assessments). The annual number of responses conducted in 2019 was estimated based on five months of data (Aug–Dec); all other results were calculated directly. **Results:** From August 2019 to December 2022, 58 HAI/AR Programs reported 141,445 responses (87% COVID-19, 11% nMDROs, 2% other HAIs or infection control breaches). Annually, programs conducted an estimated 5,546 responses in 2019, and this figure rose to 42,359 in 2020, 49,124 in 2021, and 47,651 in 2022. Outbreak responses involved 110 different pathogens, including emerging infectious diseases (e.g., SARS-CoV-2, mpox), nMDRO (e.g., carbapenemase-producing organisms, Candida auris), and other pathogens (e.g., hepatitis viruses, Mycobacterium abscessus) across >20 setting types (e.g., acute care hospitals, skilled nursing facilities, ambulatory surgery centers, assisted living facilities). Additionally, programs responded to infection control breaches in the absence of identified patient infections, including drug diversion, medical device reprocessing, and injection safety breaches. Programs conducted 50,245 infection control assessments during reported responses. **Conclusion:** From 2019–2022, as the COVID-19 pandemic took hold, HAI/AR Programs effectively utilized CDC funding to scale their response operations with an 8-fold increase in annual response activity, including a 24% increase for non-COVID-19 responses. Programs adapted responses to various pathogens, including emerging infectious diseases, across various setting types. Health department staff utilized technical expertise to conduct infection control assessments. This analysis provides valuable insights into the resilience and impact of HAI/AR Programs nationwide.

